# Tissue-type mapping of gliomas

**DOI:** 10.1016/j.nicl.2018.101648

**Published:** 2018-12-25

**Authors:** Felix Raschke, Thomas R. Barrick, Timothy L. Jones, Guang Yang, Xujiong Ye, Franklyn A. Howe

**Affiliations:** a Institute of Radiooncology - OncoRay, Helmholtz-Zentrum Dresden-Rossendorf, Rossendorf, Germany; b Neurosciences Research Centre, Molecular and Clinical Sciences Research Institute, St. George's, University of London, Cranmer Terrace, London SW17 0RE, UK; c Atkinson Morley Department of Neurosurgery, St George's University Hospitals NHS Foundation Trust, Blackshaw Road, London SW17 0QT, UK; d National Heart and Lung Institute, Imperial College London, London SW7 2AZ, UK; e Laboratory of Vision Engineering, School of Computer Science, University of Lincoln, Lincoln LN6 7TS, UK

**Keywords:** Magnetic resonance spectroscopy (MRS), Multimodal MRI, Glioma, Nosologic imaging, Pattern recognition, tCho, total cholines, tCr, total creatines, CSF, cerebrospinal fluid, FLAIR, fluid attenuated inversion recovery, GII, grade II, GIII, grade III, GIV, grade IV, Glx, glutamate & glutamine, GM, grey matter, MET, metastasis, mMRI, multimodal MRI, MRS, magnetic resonance spectroscopy, MRSI, magnetic resonance spectroscopic imaging, NAA, *N*-acetyl aspartate, Ne, necrosis, PDD, probability density distribution, PDw, proton density weighted, PDn, proton density normalised, PRESS, point resolved spectroscopy, RGB, red green blue, ROI, region of interest, T2w, T2 weighted, T2n, T2 normalised, VO, vasogenic oedema, WM, White matter

## Abstract

**Purpose:**

To develop a statistical method of combining multimodal MRI (mMRI) of adult glial brain tumours to generate tissue heterogeneity maps that indicate tumour grade and infiltration margins.

**Materials and methods:**

We performed a retrospective analysis of mMRI from patients with histological diagnosis of glioma (*n* = 25). ^1^H Magnetic Resonance Spectroscopic Imaging (MRSI) was used to label regions of “pure” low- or high-grade tumour across image types. Normal brain and oedema characteristics were defined from healthy controls (*n* = 10) and brain metastasis patients (*n* = 10) respectively. Probability density distributions (PDD) for each tissue type were extracted from intensity normalised proton density and T_2_-weighted images, and *p* and *q* diffusion maps. Superpixel segmentation and Bayesian inference was used to produce whole-brain tissue-type maps.

**Results:**

Total lesion volumes derived automatically from tissue-type maps correlated with those from manual delineation (*p* < 0.001, *r* = 0.87). Large high-grade volumes were determined in all grade III & IV (*n* = 16) tumours, in grade II gemistocytic rich astrocytomas (*n* = 3) and one astrocytoma with a histological diagnosis of grade II. For patients with known outcome (*n* = 20), patients with survival time < 2 years (3 grade II, 2 grade III and 10 grade IV) had a high-grade volume significantly greater than zero (Wilcoxon signed rank *p* < 0.0001) and also significantly greater high grade volume than the 5 grade II patients with survival >2 years (Mann Witney *p* = 0.0001). Regions classified from mMRI as oedema had non-tumour-like ^1^H MRS characteristics.

**Conclusions:**

^1^H MRSI can label tumour tissue types to enable development of a mMRI tissue type mapping algorithm, with potential to aid management of patients with glial tumours.

## Highlights

• Non-Gaussian multimodal MRI characteristics of high and low grade glioma tissue.
• Bayesian inference of multimodal MRI derives whole brain tumour tissue-type maps.
• Automated segmentation of normal and tumour tissue volumes.
• Visualisation of glioma heterogeneity, infiltration, necrosis and vasogenic oedema.

## 1 Introduction

Gliomas are the most common primary brain tumour and have heterogeneous patterns of infiltrative growth (Burger et al., 1988). Determination of glioma grade, identifying viable tumour core and infiltration margins are paramount for optimal patient management. Histopathological grading and genetic phenotyping of targeted surgical biopsy determines first line treatment (Weller et al., 2017), however this cannot provide spatial information which is essential for subsequent radiotherapy planning and to assess treatment response. While conventional MRI does indicate likely tumour grade (Lasocki et al., 2015), it fails to determine the extent of tumour infiltration into surrounding normal appearing brain (Yamahara et al., 2010). This is reflected in current radiotherapy treatment guidelines, recommending fixed irradiation margins of 2-3 cm beyond T1-weighted contrast enhancement for high-grade gliomas (Stupp et al., 2009) that aims to also irradiate and treat any infiltrative tumour tissue that is present. Consequently, such generalised radiation margins can result in “overtreatment” of healthy brain tissue, under treatment of tumour infiltration and hamper urgently needed dose-escalation strategies targeted to the macroscopic tumour volume rather than a generalised clinical target volume. These current shortcomings in high grade glioma treatment are illustrated by the fact that almost all high grade gliomas recur, with around 80% showing recurrence in the high dose fields and 20% showing more distant recurrence associated with tumour infiltration beyond this clinical target volume (Minniti et al., 2010).

Techniques such as perfusion weighted imaging (PWI) (Akbari et al., 2016), diffusion tensor imaging (DTI) (Price et al., 2006), and 1H magnetic resonance spectroscopy (MRS) (Ken et al., 2013; Raschke et al., 2015; Stadlbauer et al., 2007) provide additional information regarding tumour grade and infiltration. Multimodal magnetic resonance imaging (mMRI) combined with pattern recognition techniques can provide nosologic images that identify likely tissue-type (Akbari et al., 2016; De Edelenyi et al., 2000; Luts et al., 2009; Verma et al., 2008). Development of mMRI classifiers requires tissue-type labelling of training data, however, expert labelling is subjective and often limited to small regions of high label confidence (Akbari et al., 2016; Verma et al., 2008). Magnetic resonance spectroscopy imaging (MRSI) has been used to create tissue-type maps both as a single modality (Ken et al., 2013; Raschke et al., 2015) and in combination with MRI (De Edelenyi et al., 2000; Luts et al., 2009). 1H MRS shows good accuracy for classifying glioma grade (Raschke et al., 2012) and MRSI has been used to map underlying tissue-type components for glial tumours (Raschke et al., 2015). However, compared to MRI, MRSI is technically difficult, has long acquisition times and does not easily provide whole brain coverage.

The goal of this study was to develop an analysis method to combine mMRI to provide tissue classification and thus determine the grade, spatial extent and heterogeneity of brain tumours. Here we present the proof-of-principle in a cohort of 25 low grade and high grade glioma patients. Our approach called tissue-type mapping, uses an expandable Bayesian analysis of mMRI data. Previous Bayesian tissue segmentation techniques have assumed Gaussian probability distributions in application to MRI data (e.g. (Zhang et al., 2001; Ashburner and Friston, 2005; Prastawa et al., 2004)), and often use spatial priors. The novel technique applied in our study is not limited by the underlying assumptions of Gaussian statistics and does not require tissue spatial priors – a particular advantage in tissue segmentation of tumours that are heterogeneous in location and presentation.

We use non-Gaussian, non-spatial priors constructed from the properties of mMRI. In an initial step, MRSI is used to objectively define regions of pure high-grade and low-grade glial tumour tissue from which the distributions of isotropic diffusion, *p*, anisotropic diffusion, *q*, proton density weighted (PDw) and T2-weighted (T2w) intensities associated with these tissue types are extracted. The image intensity distributions within this 4D space are used to provide prior tissue-type probability density distributions (PDDs) for input to a Bayesian classification scheme that generates whole brain tissue probability maps. We used isotropic, *p* and anisotropic, *q* diffusion maps as input data for the tissue-type technique as they have been shown to identify tumour infiltration **(**Price et al., 2006**;**Lu et al., 2004**)**. Furthermore, in this pilot study we also use intensity normalised proton density weighted (PDn) and T2-weighted (T2n) images as input data, as they represent surrogates of fully quantitative MRI. In this study we demonstrate the potential of our tissue-type mapping technique to identify tumour grade and its spatial distribution and investigate the relationship between high-grade tissue volumes identified by the tissue-type mapping technique and patient survival.

## 2 Methods

### 2.1 Subjects

35 brain tumour patients with histopathological diagnosis (mean age 53 ± 13 years) were studied retrospectively: WHO grade II (5 diffuse astrocytomas, 3 gemistocytic rich astrocytomas, 1 oligoastrocytoma); WHO grade III (3 anaplastic astrocytoma, 1 anaplastic oligoastrocytoma); WHO grade IV (11 glioblastomas, 1 gliosarcoma); and 10 brain metastases (MET). (Note: these subtypes reflect histopathological terminology at the time of original diagnosis, whereas in the current WHO 2016 classification (Louis et al., 2016), what may have been defined as oligoastrocytoma is now defined as either astrocytoma or oligodendroglioma according to 1p19q status). Patients were newly diagnosed and treatment naive. The 2D MRSI data of the 25 glioma patients has been previously analysed (Raschke et al., 2015; Yang et al., 2015b). We used previous results (Raschke et al., 2015) to select MRSI voxels corresponding to pure tumour (see Section 2.4.) in the current study. MRI data for characterisation of healthy brain tissue was obtained from a subset of 10 age-matched individuals that were previously scanned using the same mMRI protocol as part of the St George's Neuroimaging in the Elderly (GENIE) study (Charlton et al., 2006). All data were obtained in accordance with local ethics procedures.

### 2.2 Magnetic resonance image acquisition and pre-processing

Multimodal MRI data was acquired on a 1.5-T Signa Horizon (GE Medical Systems, Milwaukee, WI, USA) equipped with 22 mT m^−1^ gradients using a quadrature head coil and comprised standard clinical MRI with the addition of DTI and 2D 1H MRSI.

Whole brain 3D T1-weighted images were acquired with a gradient echo with field of view (FOV) 240 mm × 240 mm × 186 mm and 0.9375 mm in-plane resolution with 1.5 mm slice thickness with data acquired pre- and post- intravenous injection of a Gd-based contrast agent.

Whole brain T2-weighted FLAIR images were acquired with FOV 220 mm × 220 mm, a 256 × 256 acquisition matrix across 29 slices with 5 mm thickness (TE = 133 ms, TR = 9000 ms, TI = 2200 ms).

PD-weighted (PDw) and T2-weighted (T2w) images were acquired using a dual spin echo sequence (TE = 14/98 ms, TR = 3500 ms) with FOV of 240 mm × 240 mm, a 256 × 256 acquisition matrix, and 29 slices with 5 mm thickness. Image intensity normalisation using histogram matching was applied across the entire dataset of T2w and PDw images to enable semi-quantitative comparisons of T2w and PDw images between patients (Verma et al., 2008). Prior to intensity normalisation the T2w and PDw images were skull stripped using Brain Extraction Tool (FMRIB software library, Oxford University, United Kingdom, https://fsl.fmrib.ox.ac.uk/fsl/, BET, (Smith, 2002)) and the intensity histograms were translated and scaled to minimise the L2 norm of the difference to a randomly chosen reference T2w and PDw histogram respectively (Verma et al., 2008; Zacharaki et al., 2009). Intensity normalised T2w and PDw images are referred to as T2n and PDn throughout this manuscript.

DTI data was acquired using a diffusion-weighted spin-echo with an echo-planar imaging sequence (TE = 80 ms, TR = 7000 ms) in 12 diffusion gradient directions with b = 1000 s mm^-2^ and four averages. Eight b = 0 s mm^-2^ images were also acquired. Contiguous whole brain coverage was obtained (50 slices 2.8 mm thick) using two interleaved acquisitions with slice spacing 2.8 mm (FOV 240 mm × 240 mm using a 96 × 96 acquisition matrix). DTI data was interpolated to a 256 × 256 matrix by zero filling to provide an in-plane voxel resolution of 0.9375 mm^2^. Motion and eddy current correction was applied and the diffusion tensor was computed at each voxel. Isotropic *p* and anisotropic *q* diffusion were used to quantify isotropic and anisotropic diffusion tensor components, respectively, and were calculated as proposed by Peña et al. (Peña et al., 2006). Data were skull stripped using BET (Smith, 2002).

MRSI data was acquired with the GE PROBE-SI protocol using PRESS localisation (TE = 30 ms, TR = 2000 ms), outer volume suppression and a full k-space 16 × 16 phase-encoded matrix for a single slice with nominal voxel size of 13.75 mm × 13.75 mm × 15 mm. MRSI data was zero filled to 32 × 32 voxels resulting in a nominal voxel size of 6.875 mm × 6.875 mm × 15 mm. Only MRSI voxels fully within the PRESS excitation volume were used for analysis using LCModel (Provencher, 2001) as described in Section 2.4.

### 2.3 Magnetic resonance image co-registration

To allow voxel-wise multimodal analysis all MRI for each patient were co-registered and resliced to the orientation and spatial resolution of the DTI data. The T2n image was co-registered and resliced to the DTI (i.e. to the image without diffusion sensitisation, b = 0 s mm^-2^) using a rigid body transform estimated using normalised mutual information in Statistical Parametric Mapping software (SPM12, http://www.fil.ion.ucl.ac.uk/spm/). The same rigid transform was applied to the PDn image, ensuring all images were aligned to the DTI data. The T1w and FLAIR images were also co-registered and resliced to the b = 0s mm^-2^ image using a rigid body transform estimated using normalised mutual information in SPM. Although FLAIR images have generally superseded dual-echo for radiological assessment (Bynevelt et al., 2001) and post-contrast T1w images are a key diagnostic modality, we extracted the prior PDDs from the *p*, *q*, T2n, and PDn images only, as a precursor to fully quantitative T2 and PD acquisitions in future studies.

### 2.4 Constructing a priori probability density distributions for each tissue type

Prior probability density distributions (PDDs) were generated from *p*, *q*, T2n, and PDn images for the following tissue-types: GIV glioma, GII glioma, GIV necrotic tissue, vasogenic oedema, normal grey matter, normal white matter and cerebrospinal fluid. Tissue-type PDDs were derived from mMRI within regions with the highest confidence of being a single tissue class. The following regions of interest (ROIs) were selected.

**Tumour tissue ROIs**: Within each MRSI voxel, LCModel was used to linearly decompose the 1H spectrum into normal brain, grade II (GII) and grade IV (GIV) components (Raschke et al., 2015). GII and GIV ROIs were automatically defined from MRSI voxels for which the spectrum was comprised of >90% GII or >90% GIV glioma components from GII and GIV gliomas respectively.
**Normal tissue ROIs**: Multichannel tissue segmentation (SPM12, http://www.fil.ion.ucl.ac.uk/spm/) was applied to T2n and PDn images of normal controls. A probability threshold of 0.95 was used to create binary maps of grey matter (GM), white matter (WM) and cerebrospinal fluid (CSF).
**Vasogenic oedema ROIs**: Manual ROIs were drawn by an expert (neurosurgeon TLJ) around visible oedema on *p* diffusion maps of the MET cases, with specific exclusion of the tumour core, using MRIcro (http://www.mccauslandcenter.sc.edu/crnl/tools).

**Fig. 1:**
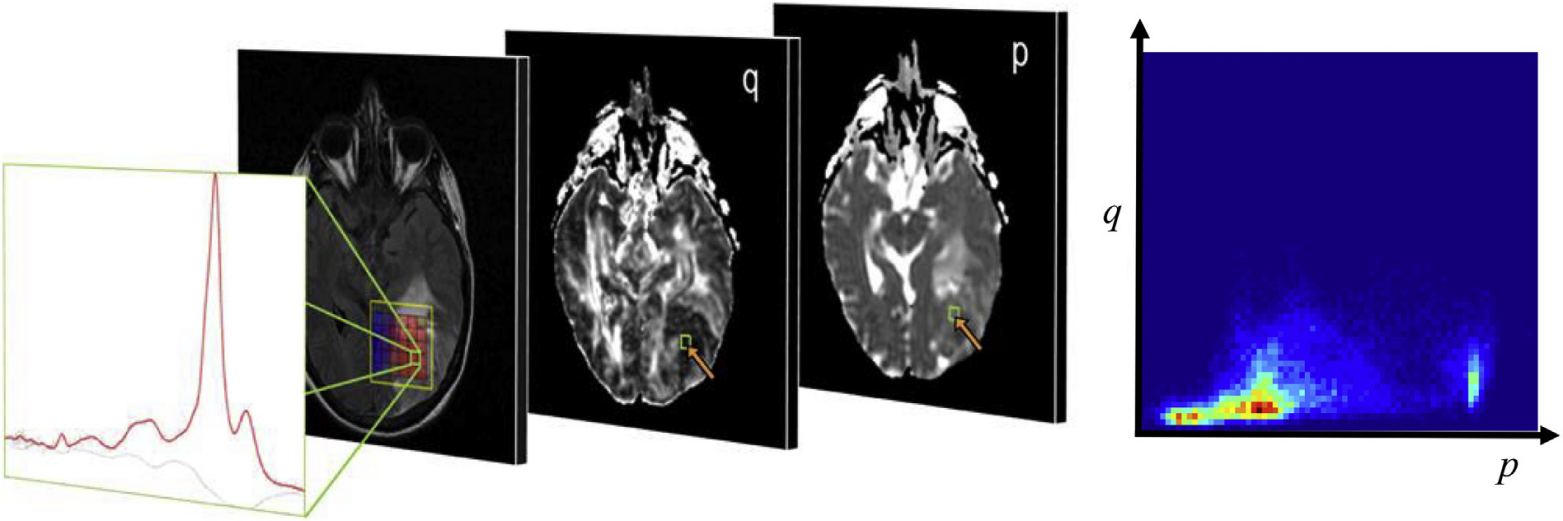
Schematic illustration showing the extraction of *p* and *q* diffusion imaging information from a region of interest defined by MRSI for a GIV voxel to create PDDs. The green ROI represents the metabolite spectrum for an MRSI voxel with pure GIV metabolic characteristics (far left). All imaging voxels within this ROI are identified on the *p* and *q* diffusion maps. Over the cohort of high-grade tumour patients the (*p*,*q*) information within all MRSI defined high-grade regions is represented as a 2D histogram distribution (far right), where red represents the highest probability density. This histogram is a 2D projection of the full 4D probability density distribution used in our classification scheme. The 2D probability density distribution (far right) contains distinct tissue sub-types of heterogeneous, but cellular, high-grade tumour, and of necrotic tissue (which has a higher magnitude of isotropic (*p*) diffusion).

**Fig. 2:**
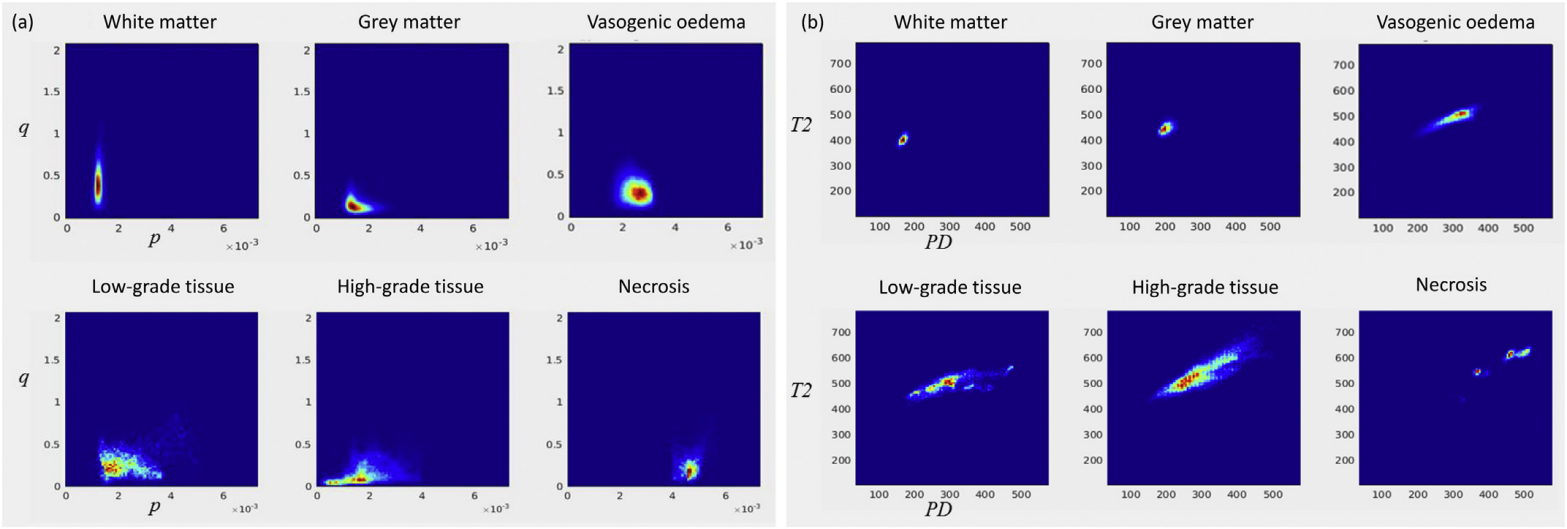
2D projections of the 4D tissue-type probability density distributions (PDDs) are shown in (a) the (*p*,*q*) plane, and (b) the (*PDn*,*T2n*) plane for each tissue type. Well-defined clusters are identified for white matter and grey matter in the (*PDn*,*T2n*) plane indicating an accurate intensity normalisation across the imaging data. High-grade tumour data is separated into sub-types of cellular tumour and necrotic tissue on the basis of the magnitude of the isotropic (*p*) diffusion parameter. The low-grade and necrosis PDDs show heterogeneous distributions with multiple clusters identified in the (*PDn*,*T2n*) PDDs that may indicate different tissue sub-tissue types.

The tissue specific ROIs described above were used to extract *p*, *q*, T2n, and PDn voxel intensities. The necrotic tissue ROIs was created by extracting mMRI data from GIV MRSI voxels with *p* > 4 × 10^−3^ mm^2^ s^-1^. The tissue PDDs were generated by mapping the mMRI ROI voxel intensities to a 4D space with axes represented by *p*, *q*, T2n, and PDn data. Each 4D histogram was normalised to unit volume with 50 bins along each axis, thus creating a 4D PDD for each tissue-type. Fig. 1 illustrates the PDD computation process for *p* and *q* data from a pure GIV voxel. PDDs for GM, WM, vasogenic oedema (VO), GII, GIV and necrotic tissue (Ne) are shown in Fig. 2 (the PDDs for CSF are not shown). The 4D tissue PDDs were used as a priori information in a Bayesian model to compute tissue probability maps.

### 2.5 Computation of tissue probability maps

**Fig. 3:**
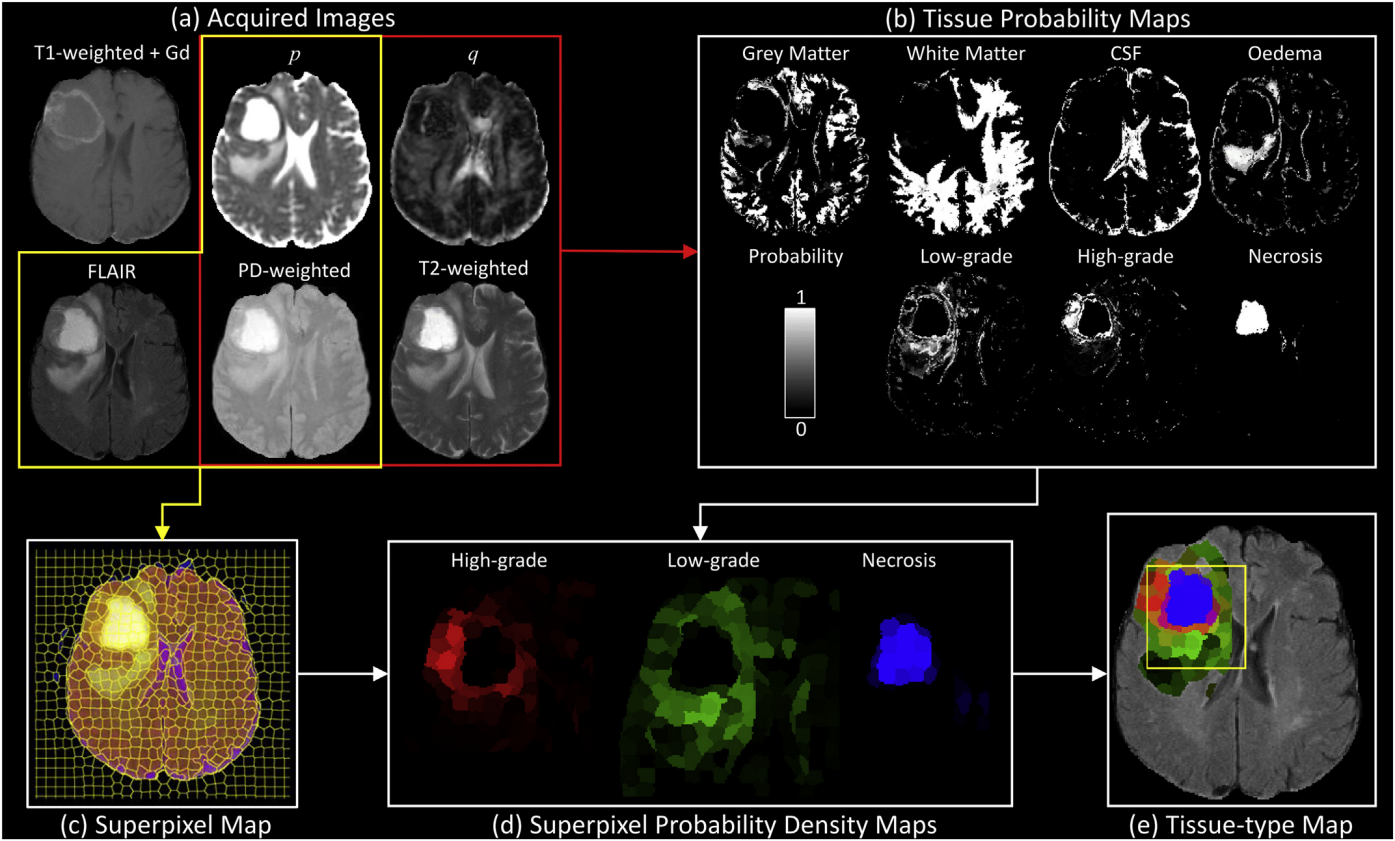
Illustration of the method for computation of tissue-type maps. (a) Data acquisition included T1w, T2w and PDw, FLAIR and diffusion tensor images (from which isotropic *p* and anisotropic *q* maps were computed). All acquired images were co-registered and resliced to the DTI space. (b) *p*, *q*, T2-weighted and PD-weighted images (red box) were used to compute voxelwise tissue probability maps by application of Bayesian statistics and tissue-type prior probability density distributions. Tissue probability maps were computed for grey matter, white matter, cerebrospinal fluid (CSF), vasogenic oedema, low-grade tissue, high-grade tissue and necrosis. (c) *p*, FLAIR and PD-weighted images (yellow box) were used to compute the superpixel map. The superpixel spatial resolution was chosen to identify major tissue boundaries. (d) Mean tissue probabilities were calculated within each superpixel and were used to identify high-grade tumour tissue (i.e. *p*(GIV)) shown in red, low-grade core or tumour infiltrated tissue (i.e. *p*(GII)) in green, and necrotic tissue (*p*(Ne)) in blue. (e) The composite RGB tissue-type colour map generated by the images illustrated in (d) is shown overlaid on the FLAIR image and provides a visual tissue-type assessment for all superpixels that contain abnormal tissue (i.e. *p*(VO) + *p*(GII) + *p*(GIV) + *p*(Ne) > 0.5). Red regions correspond to high-grade tissue, green regions to low-grade or tumour infiltrated tissue, blue regions to necrotic tissue and black regions to vasogenic oedema. A colour wheel showing all possible tissue-type colours is illustrated in Fig. 4.

Bayes' theorem was used to calculate the posterior probability *P*(*C*_*i*_*|X*) of each voxel intensity *X,* belonging to tissue class *C*_*i*_ where *i∈{1*, …*,7}* using,

(1) $$P\left(C_{i}|X\right)=\frac{P\left(C_{i}\right)p\left(X|C_{i}\right)}{\sum_{j}P\left(C_{j}\right)p\left(X|C_{j}\right)},$$

where *P*(*C*_*i*_) is the a priori probability of a voxel intensity *X,* belonging to tissue class *C*_*i*_*,* and *p*(*X|C*_*i*_) is the probability of tissue class *C*_*i*_ for a specific value of *X*. In this study, *X* = (*x*_*1*_, *x*_*2*_, *x*_*3*_, *x*_*4*_) is a feature vector comprising the four mMRI intensities (i.e. *p*, *q*, T2n & PDn, see red rectangle in Fig. 3a). The a priori probabilities were calculated from the 4D tissue PDDs as follows,

(2) $$P\left(C_{i}\right)=\frac{p\left(X|C_{i}\right)}{\sum_{j}p\left(X|C_{j}\right)}$$

Tissue probability maps (Fig. 3b) were computed for all tumour patients on a voxel-wise basis using the data from the four image modalities.

### 2.6 Superpixel segmentation – Identification of local homogeneous tissue regions

Superpixels are polygonal shaped regions for which the image voxels within them have similar imaging characteristics and can be used to aid tissue-type classification (Soltaninejad et al., 2017). Here they are used to provide local averaging of the probability maps to reduce the presence of spurious tissue-type classifications arising from noise. Superpixels were created using the Simple Iterative Clustering (SLIC) algorithm (Achanta et al., 2012) implemented as jSLIC (Borovec and Kybic, 2014) in Fiji ((Schindelin et al., 2012), http://fiji.sc/) where the minimisation function includes spatial and intensity information across three image channels. We selected PDn, FLAIR, and *p* images as the minimum dataset that gave a segmentation with visually distinct tissue-types within each superpixel. FLAIR was included to ensure the superpixel segmentation correctly distinguished between CSF and oedema. The *q* diffusion image was excluded because of its heterogeneity within white matter. Prior to segmentation the image data was interpolated to a 1024 by 1024 matrix, and the default jSLIC values of grid-size 30 and regularisation parameter 0.2 were used, which ensured the superpixel contours followed the major normal and pathological tissue boundaries. The superpixel mesh was then mapped onto the raw probability maps (Fig. 3c) from which the average tissue type was calculated within each superpixel.

### 2.7 Tissue-type colour maps

Probabilities provided by the voxelwise tissue probability maps (Fig. 3b) were averaged within each superpixel (Fig. 3c) and normalised within each superpixel to sum to 1. These tissue-type probabilities were used to automatically segment the entire lesion using the following inequality,

(3) $$p\left(\mathrm{VO}\right)+p\left(\mathrm{GII}\right)+p\left(\mathrm{GIV}\right)+p\left(\mathrm{Ne}\right)>0.5,$$

and extracting the largest connected 3D superpixel component. Superpixels were then coloured according to a RGB colour scheme (Fig. 3d) to identify high-grade cellular dense tissue (red), low-grade core and tumour infiltrated tissue (green), necrotic tissue (blue) or vasogenic oedema (black). Colour channels were assigned according to:

(4) $$\begin{array}{l} \mathrm{Red}:\;p\left(\mathrm{GIV}\right)\\ \text{Green}:p\left(\mathrm{GII}\right)\\ \text{Blue}:p\left(\mathrm{Ne}\right) \end{array}$$

**Fig. 4:**
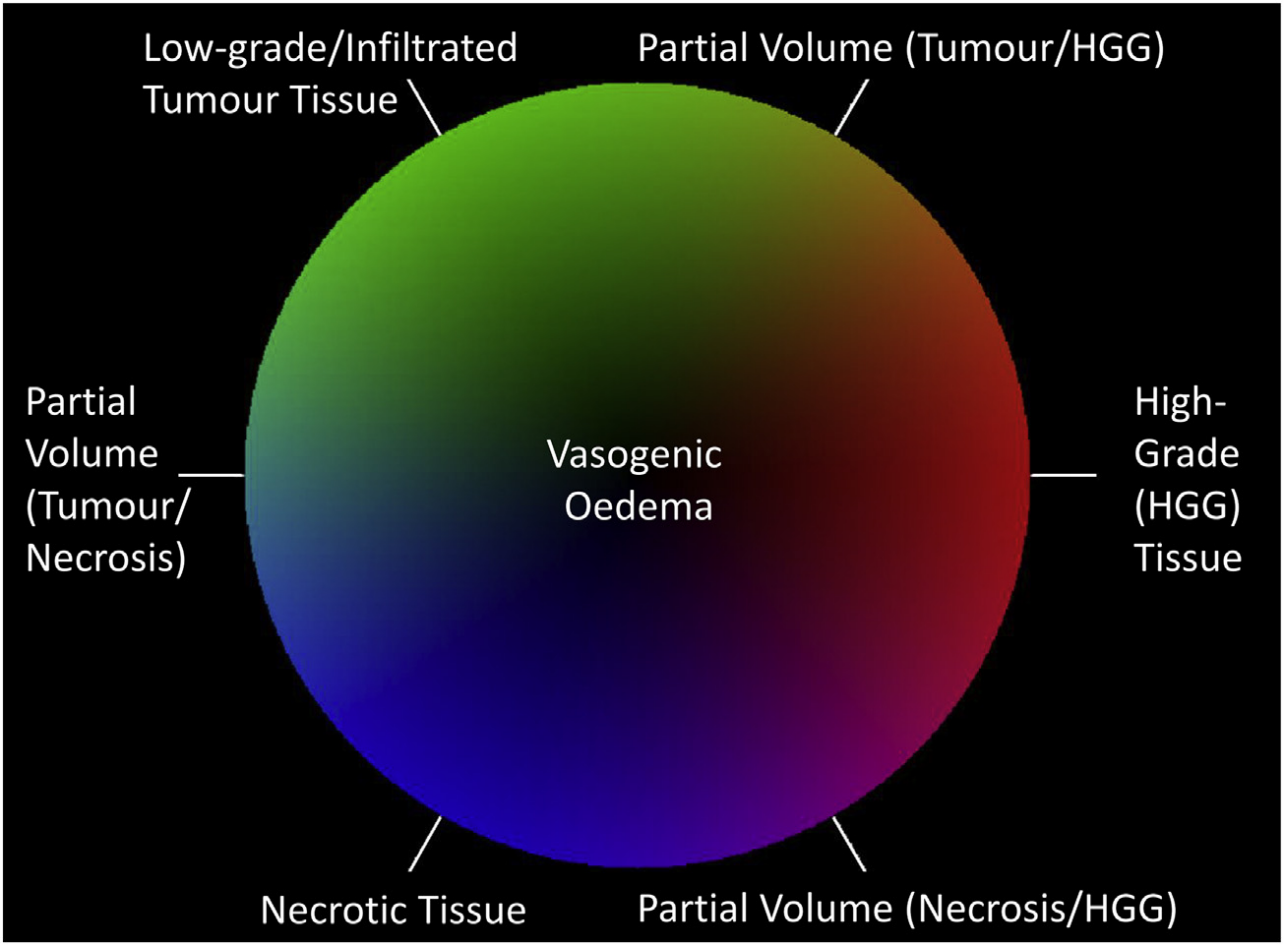
Colour wheel indicating the tissue types and partial volume information represented by the tissue-type colour maps. Red represents high-grade tissue, green - low-grade or infiltrated tissue, blue – necrotic tissue, and black – vasogenic oedema. Partial volume voxels are represented as colour mixtures such that yellow indicates partial volume of high-grade and low-grade/infiltrated tissue, magenta – partial volume of high-grade and necrotic tissue, and cyan – partial volume of low-grade/infiltrated tissue and necrosis. The colour scheme is consistent with low- and high-grade core tissue having distinct colour coding since their mMRI characteristics are different, whereas tissue infiltrated by high-grade tumour becomes indistinguishable in mMRI characteristics from low-grade tumour at low tumour partial volumes.

Resulting colours were normalised to unit length and intensity modulated according to the probability of presence of tumour tissue (i.e. *p*(*GII*) + *p*(*GIV*) + *p*(*Ne*))*.* The modulation technique ensures regions of vasogenic oedema appear black in the colour map. An example tissue-type colour map is shown in Fig. 3e. The colour wheel associated with our tissue-type mapping and its interpretation is illustrated in Fig. 4.

### 2.8 Evaluation of the tissue-type mapping technique

The results of this study were evaluated in three ways to assess their validity:

i. The tissue proportions estimated by the mMRI tissue-type technique were directly compared to those obtained by 1H MRSI using LCModel tissue type analysis (Raschke et al., 2015). To enable direct voxel-by-voxel comparison of tissue-type probabilities as determined by MRI and MRS the voxelwise tissue probability maps (Fig. 3b) were resampled to match the MRSI location and resolution.
  Tissue probabilities for non-tumorous brain (*p*(*GM*) + *p*(*WM*) *+ p*(*CSF*) *+ p*(*VO*)), low-grade tissue (*p*(*GII*)), and high-grade tissue (*p*(*GIV*) *+* *p*(*Ne*)) computed from the tissue probability maps were compared with the LCModel MRS decomposition into normal brain, GII and GIV components. The Distance Correlation technique (Székely et al., 2007) was used to investigate the relationship between these two 3D spaces (i.e. the two subspaces with axes represented by normal brain, low-grade and high-grade tissue values). Statistical analysis was performed after exclusion of the training MRSI voxels (i.e. without the mMRI data that were used to generate the a priori tumour tissue PDDs).
ii. Total lesion volume calculated from manually drawn margins on FLAIR images was compared with the abnormality volume automatically delineated by the tissue-type mapping technique. Comparison of ROI volumes was performed using Pearson's correlation with systematic volume differences assessed using a Bland-Altman analysis. Similarities between the ROIs were evaluated using the Dice coefficient, Jaccard index and Overlap coefficient.
iii. The tissue-type mapping technique provides the volume of high-grade tumour tissue within a lesion according to a given probability threshold. To evaluate the technique we compared the estimated high-grade tissue volumes extracted using a probability threshold of *p*(GIV) > 0.5 with the clinically defined grade from routine histology and patient survival.

## 3 Results

### 3.1 Probability density distributions

Fig. 2 shows 2D projections from the 4D PDDs computed from tissue specific ROIs for WM, GM, VO, GII, GIV and Ne tissue types. In (*p*,*q*) diffusion space (Fig. 2a), PDDs are non-Gaussian except for WM and VO and within healthy tissues the PDDs show less heterogeneity than in tumour tissue. The VO PDD has greater *p* and *q* diffusivity compared to tumour tissue. In (*T2n*,*PDn*) space (Fig. 2b) the GM and WM distributions are well-defined and clearly separated, confirming accurate tissue identification and effectiveness of *T2w* and *PDw* intensity normalisation across subjects. Abnormal tissue types are characterised by increased *PDn* and *T2n* compared to GM and WM, with clusters that may represent abnormal tissue subgroups within each tumour grade.

### 3.2 Tissue-type maps and comparison with MRSI

**Fig. 5:**
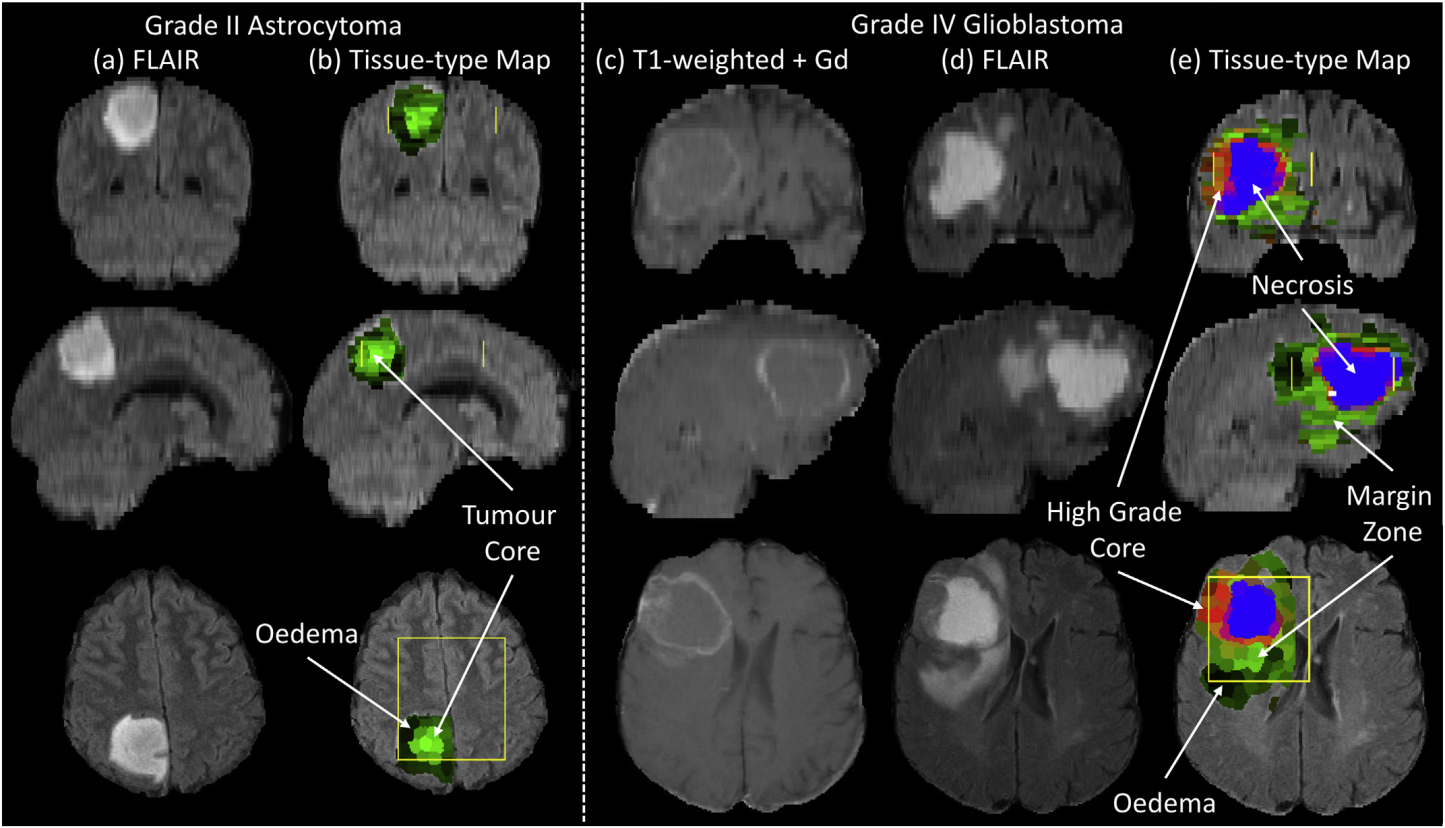
Whole brain 3D tissue-type maps of a grade II astrocytoma and grade IV glioblastoma. The astrocytoma example shows (a) FLAIR and (b) tissue-type colour maps in coronal, sagittal and axial slices through the tumour. The tissue-type colour map shows the extent of the tumour and identifies vasogenic oedema (black) surrounding a low-grade tissue tumour core (green). The margins of MRSI data acquisition are shown by the yellow lines on the tissue-type colour maps. The glioblastoma example shows (c) T1-weighted + Gadolinium contrast agent, (d) FLAIR and (e) tissue-type colour maps. The tissue-type map shows the extent of the tumour abnormality and includes the T1-weighted and FLAIR abnormality. With the proposed colour scheme, regions of tumour infiltration are represented as green, with regions exhibiting high-grade tumour features in red, necrosis in blue and vasogenic oedema as black. In (e) the whole of the green margin zone must be interpreted as tumour infiltration arising from a high-grade core.

Whole brain tissue-type colour maps for a GII astrocytoma and a GIV glioblastoma are shown in Fig. 5. A low-grade tumour core surrounded by vasogenic oedema is identified for the GII astrocytoma case. The tissue-type map provides a 3D segmentation that identifies a whole lesion boundary similar to the edge of the FLAIR abnormality region both within and beyond the limit of the PRESS MRSI region from which the tumour PDDs were initially sampled. Within the glioblastoma, a high-grade tumour core (red) with necrosis (blue) surrounded by tumour infiltrated tissue (green) and vasogenic oedema (dark) is found. The mMRI defined high-grade tumour core region includes regions of T1-weighted contrast enhancement, although contrast enhanced MRI was not used in developing our tissue-type mapping technique. Apparent tumour infiltration (green) extends beyond the FLAIR hyperintensities visible in the coronal and sagittal slices (Fig. 5d-e).

**Fig. 6:**
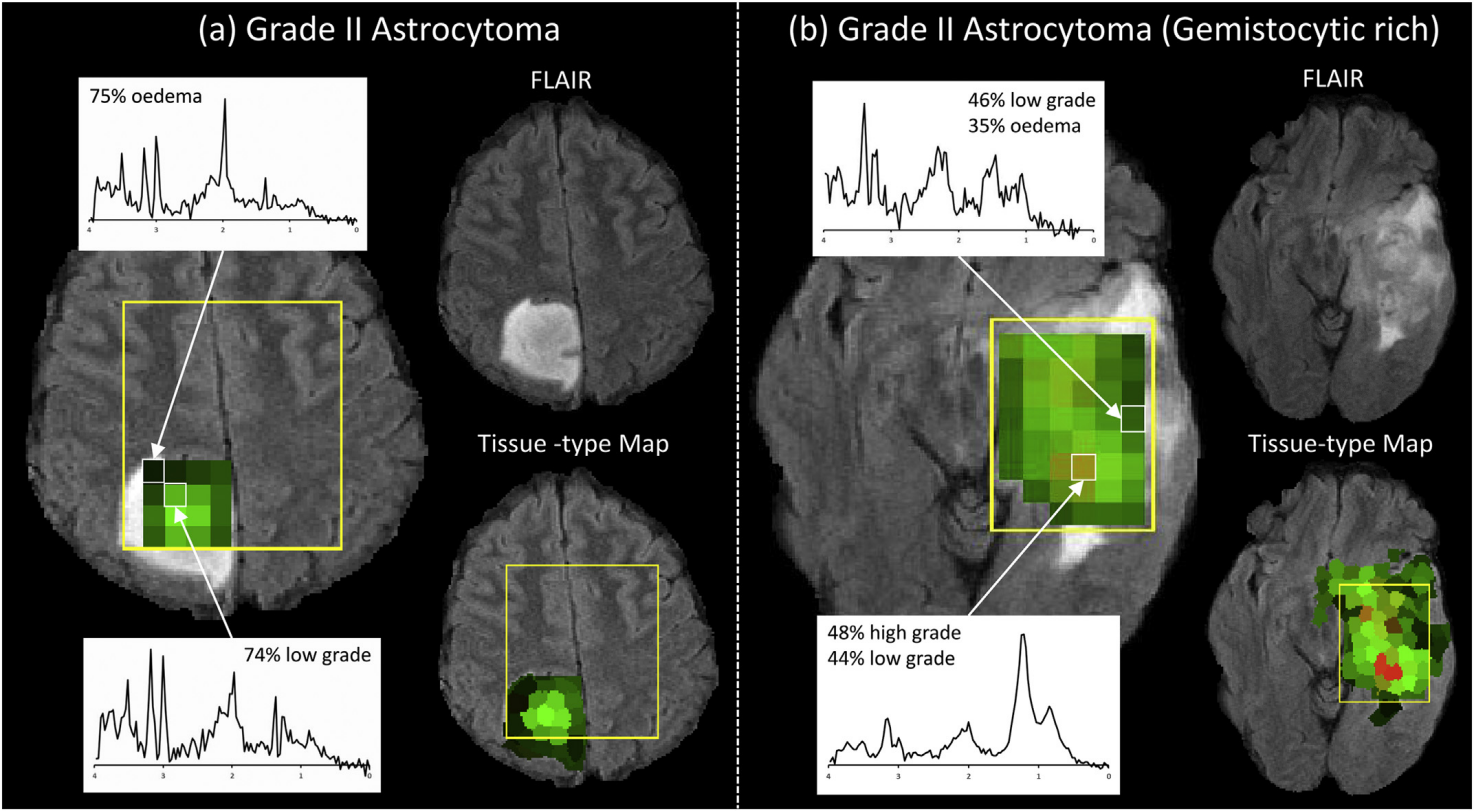
Comparison of tissue-type colour maps and MRSI data for (a) a GII astrocytoma and (b) a GII gemistocytic rich astrocytoma. MRSI spectra are shown for voxels with predominantly low-grade, high-grade and vasogenic oedema as identified from probabilities computed by the MRI tissue-type analysis after resampling to the MRSI grid. Tissue-type probabilities are given for each illustrated metabolite spectrum. The tissue-type colour map is applied to the MRSI voxel grid and represents the mean tissue-type probabilities computed within each MRSI voxel. The corresponding FLAIR images and MRI tissue-type colour maps are shown at superpixel resolution.

**Fig. 7:**
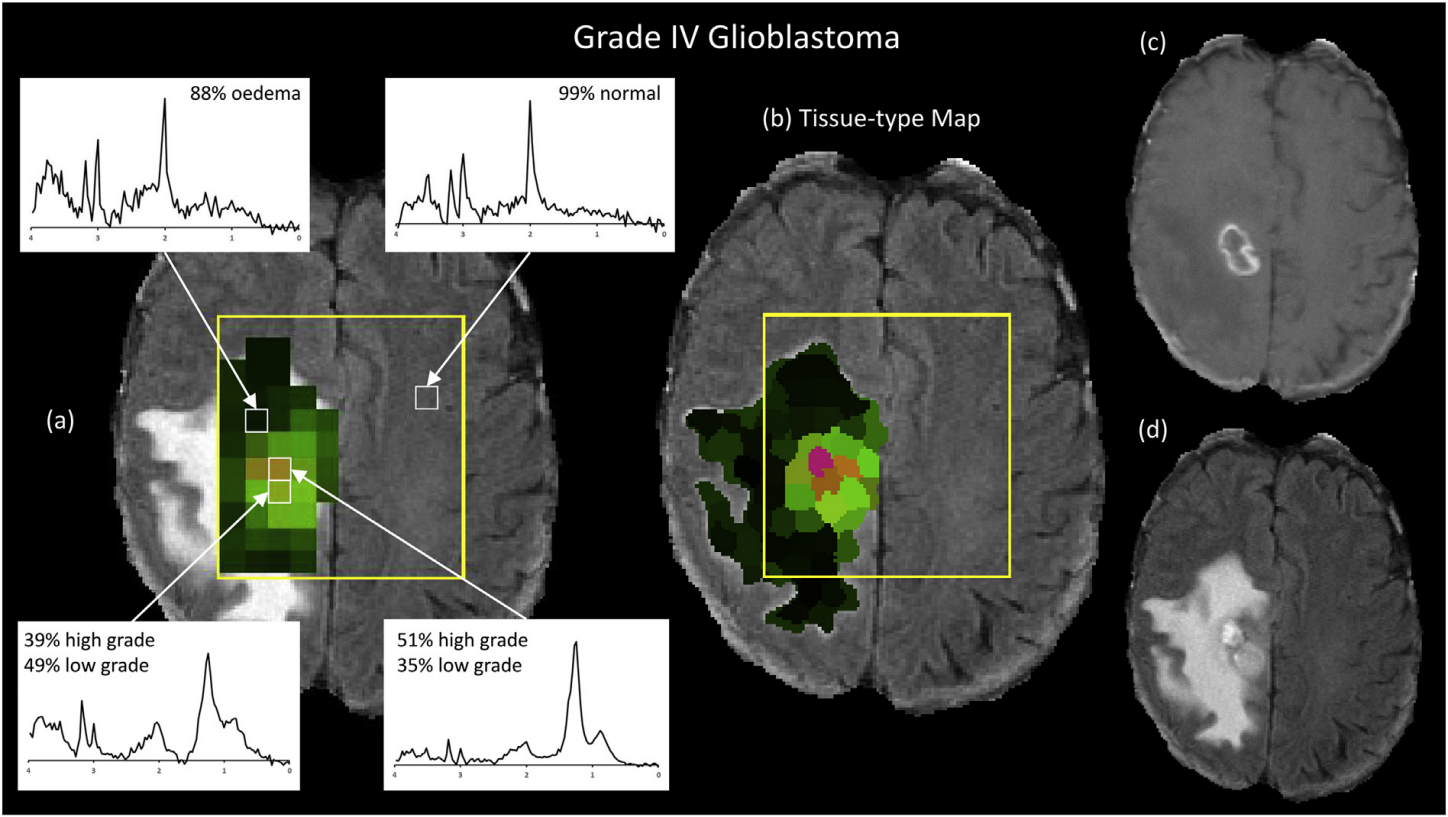
Comparison of tissue-type colour maps and MRSI spectra for a grade IV glioblastoma. Panel (a) shows MRSI spectra for voxels with normal, oedematous, low-grade and high-grade features as identified from probabilities computed by the MRI tissue-type analysis. The MRSI voxel grid is coloured based on the mean tissue-type probabilities computed within each MRSI voxel. Mean tissue-type probabilities are indicated as percentages for each spectrum. Panel (b) shows the MRI tissue-type map at superpixel resolution. Panel (c) shows the corresponding contrast enhanced T1-weighted (CE-T1w) image and (d) shows the FLAIR image. The region of contrast enhancement corresponds to the location of high-grade spectra and high-grade tissue identified by the tissue-type analysis in panel (b). Note that the superpixel tissue-type classification technique did not include data from the CE-T1w image.

Fig. 6, Fig. 7 illustrate the tissue-type segmentation and its relationship to 1H spectra within the PRESS MRSI localisation region for a GII astrocytoma (Fig. 6a), GII gemistocytic astrocytoma (Fig. 6b) and a GIV glioblastoma (Fig. 7). The tissue-type map generated from mMRI data identifies regions of vasogenic oedema, low-grade and high-grade tumour tissue that are consistent with the associated MRSI spectra. High-grade tumour tissue regions are identified in the GII gemistocytic astrocytoma and GIV glioblastoma consistent with regions of T1-weighted contrast enhancement and high-grade tissue 1H spectra. For comparison, tissue-type maps were also calculated at MRSI resolution and MR spectra of selected voxels that were not used for PDD development are shown.

**Fig. 8:**
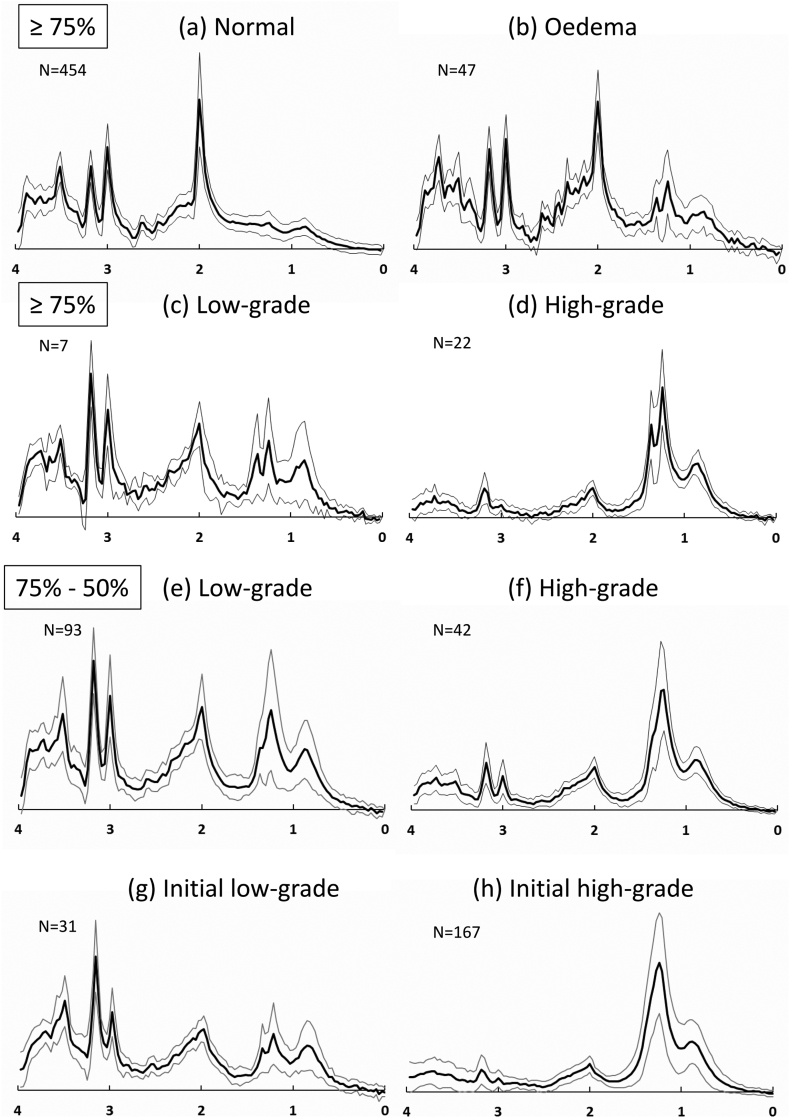
Average MRSI spectra categorised according to the MRI tissue-type analysis (a-f) and from regions used for computing prior MRI probability density distributions for Bayesian statistical analysis (g,h). Mean and standard deviations are shown for each tissue-type. (a) Normal tissue spectrum for all MRSI voxels where *p*(GM) + *p*(WM) + *p*(CSF) > 0.75. (b) Vasogenic oedema spectrum (i.e. all MRSI voxels where *p*(VO) > 0.75). (c) Low-grade tissue spectrum (i.e. all MRSI voxels where *p*(GII) > 0.75). (d) High-grade tissue spectrum (i.e. all MRSI voxels where *p*(GIV) > 0.75). (e) Low-grade tissue spectrum where 0.75 > *p*(GII) > 0.5. (f) High-grade tissue spectrum where 0.75 > *p*(GIV) > 0.5. Spectra (g) and (h) show average spectra for the MRSI voxels used to identify the initial imaging probability density distributions (priors) within the tissue-type analysis for (g) low-grade and (h) high-grade tissue. MRSI spectra included in (g) and (h) are not included in spectra (a) to (f).

Fig. 8 shows average 1H spectra identified by mean tissue-type probabilities computed within each MRSI voxel. In Fig. 8(a-d) the average spectra are taken from MRSI voxels with >75% tissue probability for: normal tissue (Fig. 8a), vasogenic oedema (Fig. 8b), and GII and GIV glioma (Figs. 8c-d). Note that MRSI voxels used to define the low-grade and high-grade tumour PDDs priors were excluded from the average spectra shown in Fig. 8 (a-f). The vasogenic oedema spectrum (Fig. 8b) has elevated glutamate plus glutamine (Glx) and the presence of lactate compared to the characteristic normal brain spectrum (Fig. 8a). Fig. 8c and d show typical tumour metabolic profiles of reduced *N*-acetyl aspartate (NAA), elevated total cholines (tCho), reduced total creatines (tCr) and the presence of lactate. The average tCho/tCr and lactate/tCr ratios are greatest in high-grade gliomas. Although the PDM analysis aims to separate viable high grade glioma tissue and necrosis, the average MRSI spectrum for high grade glioma (Fig. 8d) will inevitably contain some partial volume effects with necrosis increasing the visible lipid and lactate proportions. For MRSI voxels with calculated tissue-type probabilities between 75% and 50% (Fig. 8e-f) the average metabolic characteristics are less well-defined in terms of distinct glioma grade. Average spectra from the MRSI voxels of low- and high-grade glioma that were used initially to calculate the mMRI tumour PDDs are shown in Fig. 8g & h, respectively. These spectra show the typical metabolic profiles for low- and high-grade tissue and were used to define the a priori PDDs as they represent tissue with a predominant tumour grade.

### 3.3 Evaluation

We investigated whether the MRI derived tissue-type probabilities computed within each MRSI voxel were related to our LCModel tissue-type classification analysis (Raschke et al., 2015). The Distance Correlation technique (Székely et al., 2007) gave correlation coefficients of *r* = 0.84 across all MRSI voxels, and *r* = 0.79 when MRSI voxels used to define the a priori tumour tissue PDDs were excluded.

**Fig. 9:**
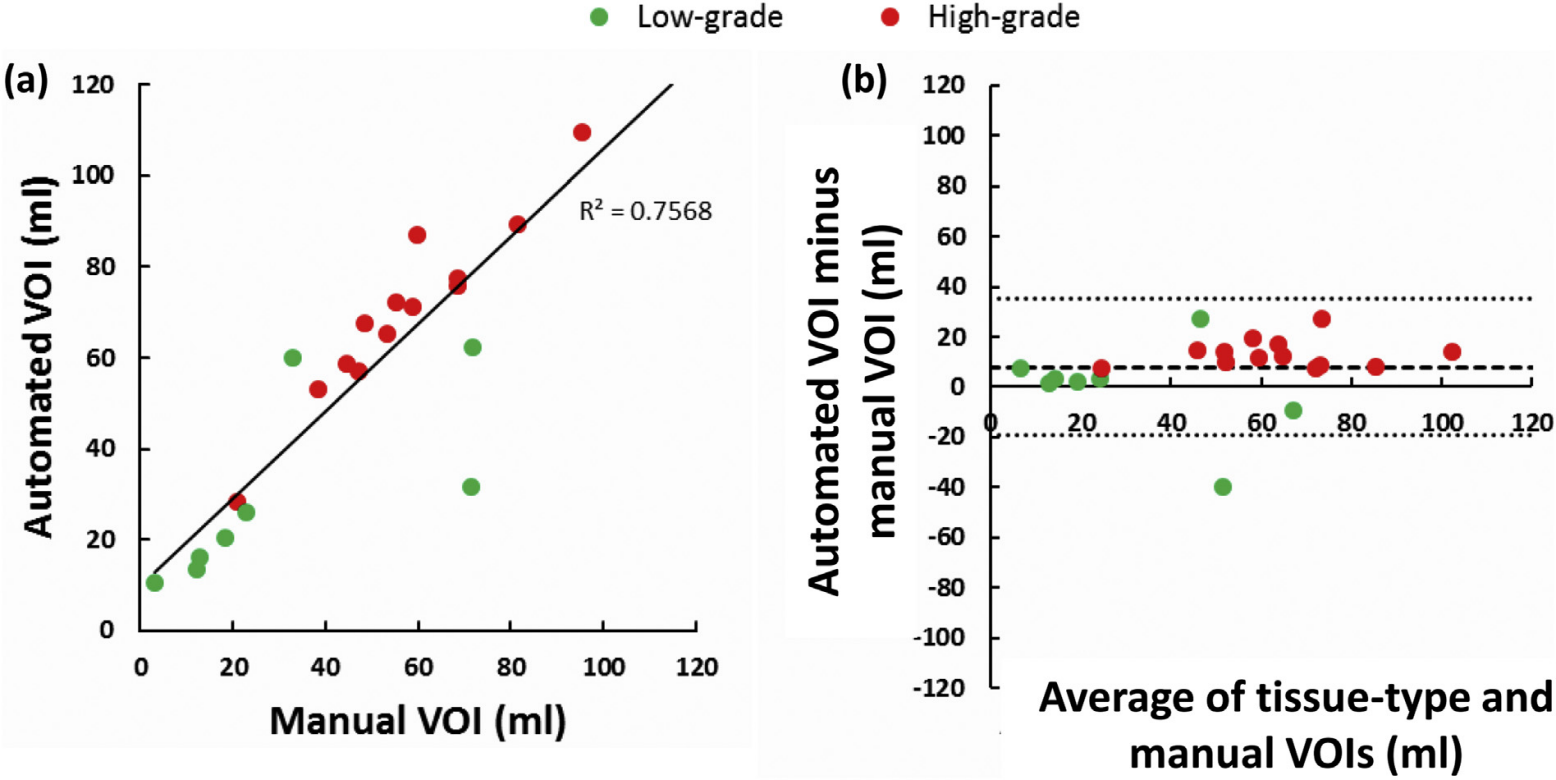
Scatterplots showing the relationship between lesion volumes (in millilitres) obtained manually by expert region drawing and automatically using the tissue-type analysis. Graph (a) shows the relationship between manual and automatic volumes. Pearson's correlation coefficient reveals a significant positive correlation between the measurements (*p* < 0.001, *r* = 0.87). Graph (b) shows a Bland-Altman plot of the same data with mean and 95% confidence limits shown. A single outlier is identified of a diffuse low-grade tumour for which the automated tissue-type volume underestimates the manual volume.

Comparison of total lesion volume from FLAIR images and the abnormality volume delineated by the tissue-type mapping show good correspondence (Fig. 9) over all tumour grades (Pearson's correlation coefficient, *p* < 0.001, *r* = 0.87). Bland-Altman analysis revealed that the automatically segmented volumes were generally larger than those determined by manual delineation, especially for high-grade gliomas (see Fig. 9b). Good correspondence between manually and automatically segmented lesion boundaries was obtained: Dice coefficient 0.77 ± 0.13, Jacard index 0.64 ± 0.14, Overlap coefficient 0.9 ± 0.08.

High-grade tissue volumes estimated from the tissue-type analysis for all superpixels with *p*(*GIV*) > 0.5 are presented according to histopathological grade in Table 1. GII astrocytoma and GII oligoastrocytoma cases are characterised by an extremely low volume of high-grade tissue (range 0–0.3 ml), but include one outlier with 49 ml of high-grade tissue. GII gemistocytic rich astrocytoma, GIII anaplastic astrocytoma and GIV glioblastoma all had comparatively greater volumes of high-grade tissue. Comparison of high-grade tumour volume with patient survival (where available) is shown in Table 2. Patient survival was ≥2 years for GII tumours, except for one GII astrocytoma (<1 year) and the two GII gemistocytic rich astrocytomas (1 to 1.5 years). GIII anaplastic astrocytoma and Grade IV glioblastoma cases were characterised by greater volumes and poorer survival (<1.5 years). Patients with survival time < 2 years (3 grade II, 2 grade III and 10 grade IV) had a high-grade tissue volume significantly greater than zero (Wilcoxon signed rank *p* < 0.0001) and volumes significantly greater than those of the 5 GII patients with survival >2 years (Mann Witney *p* = 0.0001). One GII astrocytoma showed contrast-enhancement (CE), but had negligible high-grade tissue volume (0.3 ml) and survival >2 years. Conversely the GII astrocytoma with poor survival (< 1 year) showed no CE but a large high-grade tissue volume (46 ml).

**Table 1:** Comparison of histopathological diagnosis and grade with automatically segmented volume (in millilitres) of high-grade tissue obtained by the tissue-type analysis. High-grade tissue is identified at a probability threshold of >50% (i.e. *p*(*GIV*) > 0.5). GII – grade II, GIII – grade III, GIV – grade IV.

Histological diagnosis (*n* = 25)	High-grade tissue volume (ml) (mean [range])
GII astrocytoma (*n* = 5)	0.15 [0,0.3] (*n* = 4), outlier 49 (*n* = 1)
GII oligastrocytoma (*n* = 1)	0
GII gemistocytic rich astrocytoma (*n* = 3)	6 [1,11]
GIII anaplastic astrocytoma (*n* = 3), GIII anaplastic oligoastrocytoma (*n* = 1)	10 [2,18]
GIV glioblastoma (*n* = 11), GIV gliosarcoma (*n* = 1)	52 [4,182]

**Table 2:** Comparison of patient survival time with automatically segmented volume of high-grade tissue and histopathological tumour grade. High-grade tissue is identified at a probability threshold of >50% (i.e. *p*(*GIV*) > 0.5). GII – grade II, GIII – grade III, GIV – grade IV.

Survival time (*n* = 20)	High-grade tissue volume (mean [range])	Histological Diagnosis
≥2 years (*n* = 5)	0.2 [0,0.3]	GII astrocytoma (*n* = 4)
		GII oligoastrocytoma (*n* = 1)
1 to 1.5 years (*n* = 7)	42 [7,88]	GII gemistocytic rich astrocytoma (*n* = 2)
		GIV glioblastoma (*n* = 4)
		GIV gliosarcoma (*n* = 1)
<1 year (*n* = 8)	45 [2182]	Grade II astrocytoma (*n* = 1)
		Grade III anaplastic astrocytoma (*n* = 2)
		Grade IV gliobastoma (*n* = 5)

## 4 Discussion

Our results indicate that mMRI signal intensities and their distributions can be used directly via a multi-dimensional Bayesian framework to grade and create tissue-type maps of glial tumours. In a single dimension of one MRI parameter there can be extreme overlap between different tissue PDDs, but a multi-dimensional framework allows cluster separation as can be observed for the low- and high-grade tumour tissue clusters in the T2n – PDn space, for which there is correlated variability but also cluster separation (Fig. 2b). Furthermore the probability functions of Eqs. (1) and (2) can be expanded to incorporate other tissue classes or imaging modalities to potentially improve classification simply by addition of new PDDs or expanding the PDD dimensions respectively.

A current general strategy is to incorporate MRSI as an addition to an mMRI protocol for aiding brain tumour characterisation (De Edelenyi et al., 2000; Luts et al., 2009). By using 1H MRS and its unique metabolic information, we more objectively label tumour regions for extraction of image parameter PDDs as compared to manual labelling (Akbari et al., 2016; Verma et al., 2008). The strong correlation of the tissue estimates obtained from the tissue-type analysis and those obtained from the LCModel tissue type analysis (Raschke et al., 2015), indicates, similar information to that is contained within MRS data can be extracted from our mMRI data. This major finding might enable us in future to create comprehensive tissue PDDs from a training set of cases with a full MRI protocol including MRSI, which could then be applied to cases who have not received a MRSI exam.

To the best of our knowledge, this study is the first to use 1H MRSI purely as a tool for tissue type labelling to enable mMRI tissue classification over the whole brain despite the initial restricted 1H MRSI coverage. Within the MRSI volumes, classification accuracy was assessed with average spectra. Voxels with mMRI derived probabilities >75% for either low- or high-grade glioma tissue had average spectra (Fig 8c,d) in good agreement with the average 1H spectra for voxels used for the original definition of the PDD (Fig 8g,h). The superpixel classification maps have more clearly defined tissue-types in their core than the MRSI voxels, which have partial volume estimates across several superpixels due the large MRSI slice thickness and since the MRSI grid will not necessarily match with actual tissue boundaries, whereas the superpixel boundaries are based on the image characteristics (Figs 6b & 7). Hence for MRSI voxels with single tissue-type probabilities of 50% to 75%, the average spectra show mixed tumour grades (Fig 8c, d). Voxels classified with probability >75% oedema do not have elevated tCho/tCr compared to normal brain (Fig. 8a,b) suggesting there is no tumour infiltration (Stadlbauer et al., 2007). The elevation of Glx could be related to excitoxicity (Ricci et al., 2007) due to tumour generated lactate diffusing into the peritumoural space.

Over the whole brain, the mMRI classification derives a total lesion volume that correlates well with manual delineation, but is slightly larger (Fig. 9). However, the lesion volume is determined by the chosen probability threshold for classifying abnormal tissue. A longitudinal study is needed to determine the optimal probability threshold for mapping tumour infiltration beyond the clinical target volume used for post-surgical radiotherapy dose planning.

There is good evidence that metabolic information from 1H MRSI has applications for tumour biopsy targeting, radiotherapy planning (Ken et al., 2013) and to aid prognosis (Neill et al., 2017). Mapping the MRI characteristics from tissue regions with specific 1H MRS profiles provides a surrogate metabolic marker in the form of a Bayesian analysis of mMRI. The results from our Bayesian model and PDD priors are also consistent with observations that diffusion and T2 parameters have potential to detect tumour infiltration (Price et al., 2006; Lu et al., 2004; Oh et al., 2005). The presence of mMRI derived high-grade tumour volume showed good agreement with overall patient survival (Table 2), albeit a prospective evaluation with a larger, longitudinal dataset is needed. High-grade mMRI characteristics were observed in all three gemistocytic tumours, in keeping with their more aggressive behaviour compared to other low-grade gliomas (Krouwer et al., 1991), and also in a non-enhancing GII astrocytoma of a patient with <2 yrs. survival. Such identified high-grade volumes may present promising treatment targets for future studies using dose escalation (Ken et al., 2013; Fitzek et al., 1999) or radiosurgery (Einstein et al., 2012).

There are limitations to this current retrospective study, which is on a small dataset, and does not use the 2016 WHO classification system with genetic subtypes (Louis et al., 2016). Specific MRI characteristics can be related to certain genetic subtypes (Macyszyn et al., 2016), and in future work our tissue type probability distributions need to reflect any variability between these subtypes. For example, our automated tissue-type segmentation could be used to provide direct input to other classification schemes that use volumetric (Naeini et al., 2013) or textural (Yang et al., 2015a) features for detecting tumour (sub)types with varied prognosis. Further work also requires validation using separate datasets for extracting the PDDs and for testing the classification and prognostic values of the methodology. Nevertheless, there are aspects of independent validation within our current analysis. GIV tissue characteristics were only extracted from GIV gliomas, whereas we detected likely high-grade regions within one GII astrocytoma, three GII gemistocytic astrocytomas, and four GIII tumours, with each of these patients showing poor survival (i.e. <1.5 yrs). In addition, we have 1H MRSI data that supports a classification of oedema in gliomas (Fig. 8b) for which the original mMRI tissue characteristics of oedema were extracted from a separate metastatic tumour data set. Further work is needed to more fully validate the predictive value of our classification scheme when it detects high-grade and low-grade regions beyond the contrast-enhanced region using longitudinal studies. Finally, we used PDn and T2n images that are only semi-quantitative and so contain mixed weighting. New MR fingerprinting methods (Pierre et al., 2016) will aid rapid acquisition for creation of T2 relaxation time and proton density maps, and this will enable rational development of protocols that would allow application of the method in multi-site trials and improve clinical applicability. In addition, information from other quantitative imaging methods, such as dynamic susceptibility contrast (DSC) MRI, which is of known value to aid tumour grading (Akbari et al., 2014), can be straightforwardly incorporated into Eq. (1) and may further improve the tissue classification accuracy.

In conclusion, a mathematically intuitive method for using mMRI to infer the likely tissue-type of glial brain tumours has been described, which can be readily expanded to include other imaging modalities and tissue-types. Tumour tissue-type mapping with multimodal MRI may aid management of patients with glial tumours by delineating viable tumour from oedema and normal brain to aid targeted biopsy, surgical planning and radiotherapy dose-planning, as well as prognosis for patient survival.

## Grant sponsors

Cancer Research-UK and Engineering and Physical Sciences Research Council Cancer Imaging Programme at the Children's Cancer and Leukaemia Group (CCLG), in association with the Medical Research Council and Department of Health (UK); Grant number: C7809/A10342 (FR) and Cancer Research UK project; Grant number: C1459/A13303 (GY). Data were obtained during the EU FP7 eTUMOUR project.

The funders had no influence on the study design, data analysis or preparation and submission of the manuscript.
